# Comparisons of Different Metabolic Syndrome Definitions and Associations with Coronary Heart Disease, Stroke, and Peripheral Arterial Disease in a Rural Chinese Population

**DOI:** 10.1371/journal.pone.0126832

**Published:** 2015-05-11

**Authors:** Jiangping Wen, Jingang Yang, Yujie Shi, Yuanbo Liang, Fenghua Wang, Xinrong Duan, Xilin Lu, Qiushan Tao, Xinxin Lu, Yaping Tian, Ningli Wang

**Affiliations:** 1 Department of Clinical Biochemistry, Chinese PLA General Hospital, Beijing, China; 2 Department of Laboratory Medicine, Beijing Tongren Hospital, Capital Medical University, Beijing, China; 3 State Key Laboratory of Cardiovascular Disease, Fuwai Hospital, National Center for Cardiovascular Diseases, Chinese Academy of Medical Sciences and Peking Union Medical College, Beijing, China; 4 Cardiovascular Disease Institute, Beijing Military Area Command General Hospital, Beijing, China; 5 Clinical and Epidemiological Research Center, the Affiliated Eye Hospital of Wenzhou Medical University, Wenzhou, Zhejiang Province, China; 6 Beijing Tongren Eye Center, Beijing Tongren Hospital, Capital Medical University, Beijing Ophthalmology and Visual Science Key Laboratory, Beijing, China; 7 Department of Laboratory Medicine, Handan 3rd Hospital, Handan, Hebei Province, China; 8 School of Public Health, Peking University, Beijing, China; INRCA, ITALY

## Abstract

**Objectives:**

We estimated the prevalence of metabolic syndrome (MetS) and compared associations of different MetS definitions with coronary heart disease (CHD), stroke, and peripheral arterial disease (PAD) in a rural Chinese population.

**Methods:**

Among 4,748 residents (2,145 men and 2,603 women) aged 30+ years in rural China from 2006 to 2007, the prevalence of MetS was estimated by using five different definitions: modified World Health Organization (WHO), Chinese Diabetes Society (CDS), the updated National Cholesterol Education Program Adult Treatment Panel III (NCEP-ATP III) for Asian-Americans, International Diabetes Federation (IDF), and Joint Interim Statement (JIS). Multivariable logistic regression analyses were implemented to estimate the association between MetS and the prevalence of CHD, stroke and PAD, respectively.

**Results:**

Prevalence of MetS in men was 11.5% (WHO), 14.8% (CDS), 32.4% (NCEP-ATP III), 27.5% (IDF) and 39.7% (JIS) and in women was 15.7% (WHO), 20.7% (CDS), 54.2% (NCEP-ATP III), 51.5% (IDF) and 54.2% (JIS), respectively. Respective ORs (95% CI) for associating MetS with CHD in men were 1.79 (1.02-3.17), 1.25 (0.69-2.26), 1.61 (1.01-2.58), 1.84 (1.14-2.96), and 1.53 (0.96-2.43). Corresponding ORs (95% CI) for stroke in men were 2.18 (95% CI 1.20 to 3.97), 2.20 (95% CI 1.25 to 3.89), 1.71 (95% CI 1.02 to 2.84), 1.30 (95% CI 0.77 to 2.23), and 1.61 (95% CI 0.97 to 2.68), respectively. In women, CHD and stroke were significantly associated with MetS using all five definitions of MetS. In addition, PAD was associated with all five MetS definitions in men, but not in women. Only hyperglycemia and BMI were significantly associated with PAD in women.

**Conclusions:**

In this rural Chinese population, the JIS, IDF and CDS criteria may not be more suitable than WHO and updated NCEP-ATPIII definitions for screening high-risk individuals and estimating the risk of CHD and stroke from MetS, especially in men.

## Introduction

Metabolic syndrome (MetS) is characterized by a clustering of cardiovascular risk factors, including hyperglycemia, raised blood pressure, elevated triglycerides, low high-density lipoprotein cholesterol, and obesity (particularly central adiposity) [1]. Since Reaven first described the concept of syndrome X (later renamed MetS) in 1988 [2], several MetS definitions have been proposed by different international organizations over the past decade, including the World Health Organization (WHO) [3], the National Cholesterol Education Program (NCEP) [4], the American Heart Association/National Heart, Lung, and Blood Institute (AHA/NHLBI) [5], the International Diabetes Federation (IDF) [6], and the Chinese Diabetes Society (CDS) [7]. Recently, a new Joint Interim Statement (JIS) was proposed by IDF and AHA/NHLBI to harmonize the definition of MetS [1]. Although the major components of these MetS definitions are similar, individual components of MetS and their diagnostic threshold values are differently focused on different definitions. The IDF, NCEP, and new JIS definitions emphasize central obesity, but the WHO definition focuses on insulin resistance.

To date, many studies indicate that MetS is associated with coronary heart disease (CHD), stroke, and peripheral arterial disease (PAD) [8–13]. However, few data exist to associate different definitions of MetS with CHD, stroke, and PAD risk. Moreover, available data are conflicting [14–19].

Compared with Western populations, the Chinese have lower serum cholesterol, lower prevalence of obesity, higher plasma glucose, and different profiles of cardiovascular disease (CVD) because of different genetic and environmental factors [20–23]. Several studies confirmed that the prevalence of MetS varies in the Chinese based on the definition used [24–27]. But only one study [25] compared the relationship of NCEP and IDF definitions with CVD in elderly Chinese people in Beijing. Unfortunately, WHO and CDS definitions, which have been proposed for screening high-risk Chinese individuals, were not considered. Most recently, a new JIS definition was proposed by IDF and AHA/NHLBI, but no population-based study has compared the relationship of the new JIS definition and previous definitions with CVD in the Chinese. Therefore, questions remain about which MetS definitions are more strongly associated with the risk of CVD in the Chinese.

In this study, we report the prevalence of MetS using five different definitions based on WHO [3], the updated NCEP-ATPIII for Asian-Americans [28], IDF [6], CDS [7], and the JIS [1] in a rural Chinese general population, who have different lifestyles, diets, levels of physical activity, and socioeconomic positions when compared these data with that from people living in urban areas of China. Furthermore, we compared the associations of these five different MetS definitions with CHD, stroke, and PAD, and explored whether individual components of MetS are more important determinants of CVD than any of these full definitions of MetS.

## Materials and Methods

### Study population

We performed a cross-sectional analysis using data from the Handan Eye Study (HES). Detailed information about the methods and procedures of this survey is available elsewhere [29]. The HES is a population-based study designed to survey eye diseases and other health-related problems in non-institutionalized, community dwelling persons aged 30 years and older in Yongnian, a rural county of Handan, which is located about 500 km south of Beijing and covers 980 km^2^. The population was approximately 830,000 in 2000. In this area, 80% of the population engages in farming, and 98% are of Han ethnicity. *Per capita* net income of rural households in this region is 3,468 Yuan (approximately 468 USD), which is similar to the average income surveyed (3,587 Yuan, 484 USD) for the People's Republic of China [30]. The study was approved by the Ethics Committee of Beijing Tongren Hospital, and the approval number was TREC2006-22. Written informed consent was obtained from all subjects. A stamp of the right forefinger was obtained from those who could not read or write, and the Ethics Committee approved this consent procedure.

**Fig 1:**
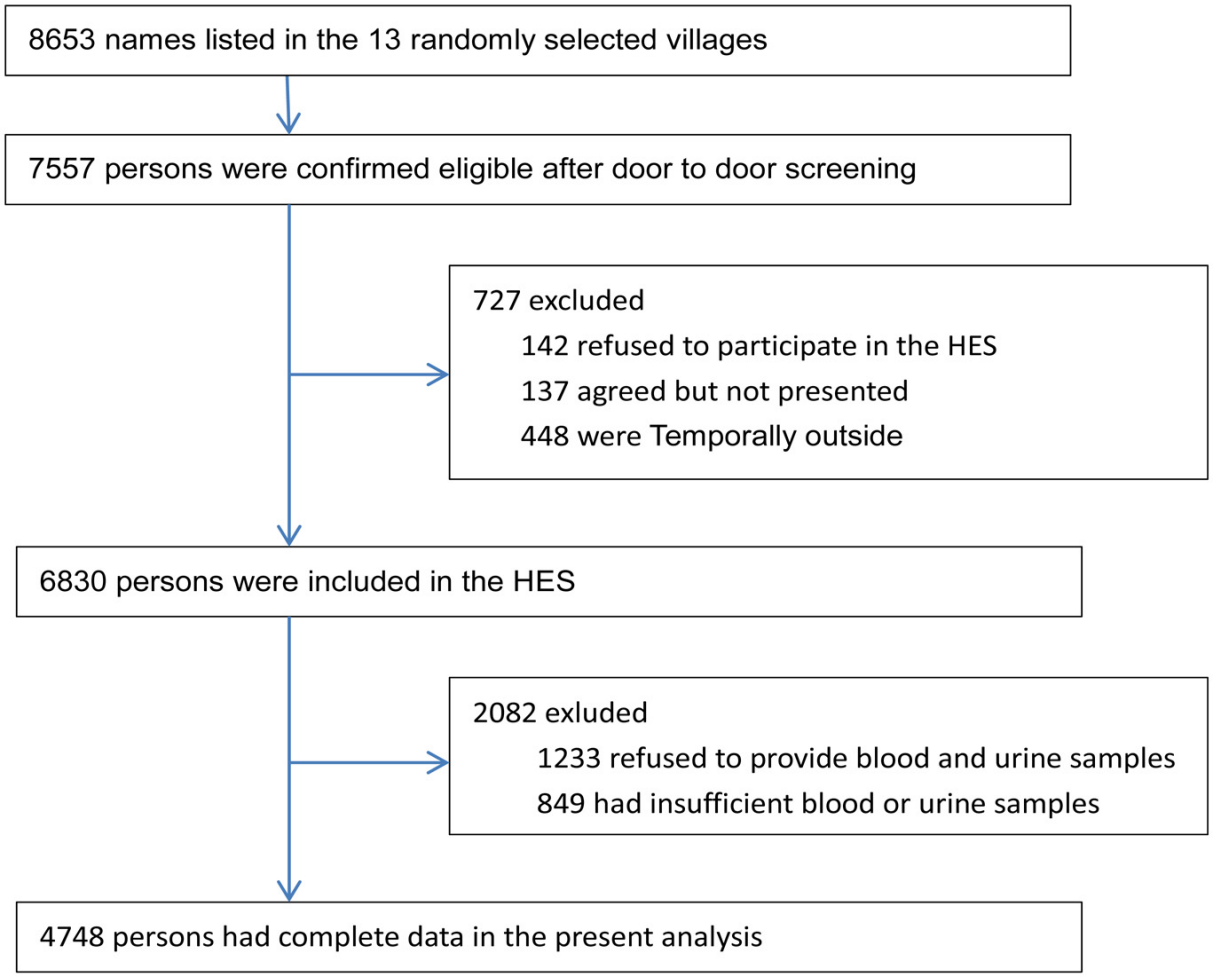
Flow diagram of recruitment of participants in the analysis.

Residents of Yongnian County aged 30 years or older were randomly selected using a cluster sampling technique, with probabilities proportionate to the size (PPS) of the population in each cluster. Of the 458 villages in Yongnian County, stratified by geographic land form (plains or hillside), 13 were selected randomly to achieve a target sample size of 5105. All residents aged 50 years and older were invited to participate in the selected 13 villages. In addition, to study individuals aged 30 to 49 years, we randomly selected 6 of the 13 villages in which all residents aged 30 to 49 years also were invited to participate. As illustrated in Fig 1, Of the 8,653 individuals screened, 7,557 were eligible for the HES. A total of 6,830 participants took part in the HES from October 2006 to October 2007. Blood and urine samples were obtained from 5,597 willing participants. After exclusion of some samples with a lack of blood volume, 4,748 had complete data for fasting plasma glucose (FPG), triglycerides (TGs), high density lipoprotein cholesterol (HDL-C), and urine albumin-to-creatinine ratio (ACR). These subjects formed the basis of the present analysis.

### Data collection

Trained interviewers used questionnaires to obtain answers about demographic information, including date of birth, gender, ethnicity, occupation, education, health status, health behavior (smoking, alcohol use, physical activity), and medical history. Smoking and alcohol use were defined as never used, current user, and former user. Participant education was categorized into 3 groups according to the number of years of education (illiterate or primary school, 0–6 years; junior high school, 7–9 years; senior high school, ≥10 years). Physical activity was classified as low (exercising rarely or never), moderate (walking or bicycling continually for more than 10 min, 1–3 times/week), and high (exercise that causes rapid respiration for more than 10 min, >3 times/week).

During the clinical examination, two blood pressure measurements were obtained with the participant in the seated position after 5 min of rest by using the noninvasive, automated Hem-907 blood pressure monitor (OMRON, Japan). Systolic blood pressure (SBP) and diastolic blood pressure (DBP) were calculated as the mean of the two independent measures. The ankle-brachial indexes (ABIs) were measured by using an ABI-form device (VP1000, Colin, Komaki, Japan), which automatically and simultaneously measures blood pressure in both arms and ankles using an oscillometric method. The ABI was calculated by the ratio of the ankle SBP divided by the arm SBP. Body height and weight were measured with subjects not wearing shoes or outerwear. Height measurements were taken with a wall-mounted measuring tape. Weight measurements were taken with a bathroom scale (RGZ-120, China). Body mass index (BMI) was calculated as weight (kg)/height (m^2^). The location of the waist measurement was set as the mid-point between the last rib and the iliac crest in the mid-axillary line.

Every participant in the study was requested to fast for at least 8 h prior to the blood drawing, which took place between 7:00 and 9:00 a.m. in the villages. FPG, TGs, and HDL-C were measured by enzymatic methods (Olympus AU2700, Japan). Urinary albumin and creatinine were tested on a morning spot urine sample. Urinary albumin was measured by immunoturbidimetric methods (Audit Diagnostic, Ireland). Urinary creatinine was measured by the Jaffé kinetic method. C-reactive protein (CRP) concentration was measured by using immuno-nephelometry with the IMMAGE immunochemistry system (Beckman Coulter, Inc., USA).

### Definition of metabolic syndrome

In the present study, we define the presence of the MetS using the following five definitions (Table 1): (1) modified WHO [3], (2) the updated NCEP-ATPIII for Asian-Americans [28], (3) IDF [6], (4) the JIS [1], and (5) CDS [7]. Because data on insulin and 2-h post load plasma glucose were not available, the WHO definition was modified slightly and described in Table 1.

**Table 1:** Definitions of the metabolic syndrome.

	Modified WHO [3]	updated NCEP-ATPIII for Asian-Americans [28]	IDF [6]	JIS [1]	CDS [7]
**Required**	FPG ≥6.1 mmol/L or known treatment for diabetes		Ethnic-based WC ≥90 cm in men and ≥80 cm in women for Chinese		
**No. of abnormalities**	And ≥2 of:	≥3 of:	And ≥2 of:	≥3 of:	≥3 of:
**HDL cholesterol**	HDL-C <0.90 mmol/L in men or <1.03 mmol/L in women	<1.03 mmol/L in men or 1.30 mmol/L in women	<1.03 mmol/L in men or 1.30 mmol/L in women	<1.03 mmol/L in men or 1.30 mmol/L in women	<0.90 mmol/L in men or <1.03 mmol/L in women
	and/or				and/or
**Triglycerides**	≥1.7 mmol/L	≥1.7 mmol/L	≥1.7 mmol/L	≥1.7 mmol/L	≥1.7 mmol/L
**Glucose**		FPG ≥5.6 mmol/L or known treatment for diabetes	FPG ≥5.6 mmol/L or known treatment for diabetes	FPG ≥5.6 mmol/L or known treatment for diabetes	FPG ≥6.1 mmol/L or known treatment for diabetes
**Obesity**	WHR >0.90 in men (>0.85 in women) and/or BMI >30 kg/m2	WC ≥90 cm in men or ≥80 cm in women		Ethnic-based WC ≥85 cm in men or ≥80 cm in women for Chinese	BMI >25 kg/m2
**Blood pressure**	≥140/90 mmHg or use of antihypertensive drugs	≥130/85 mmHg or use of antihypertensive drugs	≥130/85 mmHg or use of antihypertensive drugs	≥130/85 mmHg or use of antihypertensive drugs	≥140/90 mmHg or use of antihypertensive drugs
**Other**	Urinary albumin creatinine ratio ≥30 mg/g				

WHO, World Health Organization; NCEP-ATPIII, National Cholesterol Education Program Adult Treatment Panel III; IDF, International Diabetes Federation; CDS, Chinese Diabetes Society; JIS, Joint Interim Statement; WHR, waist/hip rate; BMI, body mass index; HDL-C, high density lipoprotein cholesterol; TGs, triglycerides; FPG, fasting plasma glucose; WC, waist circumference.

### Definition of CHD, stroke, and PAD

In the present study, CVD was defined by the presence of one or more of the following 3 outcomes: CHD, stroke and PAD. Myocardial infarction was diagnosed by a representative set of electrocardiogram, cardiac enzyme values, and typical symptoms. Angina was defined as use of nitroglycerine, experience of typical chest pain, and electrocardiogram changes compatible with ischemic heart disease. Stroke was diagnosed by typical symptoms and computed tomography. This information was gathered on interview questions at the time of the survey and verified from local hospital records. The PAD was defined as an ABI <0.9 in either leg [31]. Participants who had history of diabetes and/or a fasting plasma glucose ≥7.0mmol/L were diagnosed with diabetes.

### Statistical analysis

Statistics analysis was performed with SPSS (Statistical Package for the Social Sciences) v.18.0. The current analysis was restricted to 4,748 subjects with complete MetS data. Baseline characteristics of participants were described separately for men and women, using means ± SD or median (interquartile range) for continuous variables, and counts and percentages for categorical variables. For a comparison of the mean values, the unpaired t-test was used. Because glucose, TGs, and albumin-to-creatinine ratios were skewed, we evaluated the significance of any differences in median values between groups using the Mann-Whitney U test. Categorical variables were analyzed by with a χ^2^ test.

Multivariable logistic regression analyses were carried out to calculate the odds ratios (ORs) and their 95% confidential intervals (CIs) for prevalent CHD or stroke or PAD (dependent variables) in relation to MetS using different definitions and single-risk factor components of these definitions. MetS is used as a dichotomous variable in these logistic regression analyses, i.e. presence or absence of MetS. Other established CVD risk factors and potential confounding variables were controlled in the regression models. Variables were age, smoking, alcohol use, education, physical activity, and family history of CHD. Because the CRP data were skewed, all CRP values were natural log-transformed to the approximate normality in regression analysis.

In supplementary analyses, we repeated the main analyses to examine the associations of all five definitions of MetS with CHD, stroke, and PAD after excluding diabetic patients

## Results

The final dataset included 4,748 participants (2,145 men and 2,603 women) aged 30–97 years who took part in the HES from October 2006 to October 2007 (Fig 1). The characteristics of the study population are shown in Table 2. Compared with men, women had higher BMI, TGs, total cholesterol, HDL-C, LDL-C, ACR, and lower WC, WHR, and less education. Men smoked (58.6% versus 0.1%) and drank more (40.4% versus 0.7%) than women. Of the women 58.9% were postmenopausal at baseline, and only one woman used hormone replacement therapy (HRT). The prevalence of hypertension, diabetes, and PAD was higher in women than in men.

**Table 2:** Baseline characteristics of participants.

	Total (n = 4748)	Men (n = 2145)	Women (n = 2603)	*P* value
**Age, years**	52±11	52±11	51±11	0.034
**BMI, kg/m** ^**2**^	24.6±3.7	24.3±3.5	24.9±3.8	<0.001
**Waist circumference, cm**	87.5±9.3	88.4±8.9	86.7±9.5	<0.001
**WHR**	0.90±0.05	0.92±0.05	0.88±0.05	<0.001
**SBP, mmHg**	138.8±21.9	138.2±21.1	139.4±22.6	0.063
**DBP, mmHg**	77.6±12.1	77.9±12.4	77.4±11.8	0.205
**Total cholesterol, mmol/L**	4.61±0.95	4.46±0.88	4.73±0.98	<0.001
**HDL-cholesterol, mmol/L**	1.28±0.29	1.23±0.28	1.32±0.29	<0.001
**LDL-cholesterol, mmol/L**	2.71±0.65	2.62±0.60	2.78±0.68	<0.001
**Triglycerides, mmol/L**	1.25 (0.89–1.82)	1.20 (0.85–1.78)	1.29 (0.93–1.84)	<0.001
**Fasting glucose, mmol/L**	5.52 (5.18–5.95)	5.53 (5.18–5.94)	5.52 (5.19–5.97)	0.231
**Albumin-to-creatinine ratio, mg/g**	8.66 (4.07–19.40)	7.60 (3.63–16.67)	9.69 (4.46–21.51)	<0.001
**hsCRP** ^**a**^	0.87 (0.37–2.23)	0.81 (0.36–2.18)	0.91 (0.38–2.28)	0.256
**Smokers, n (%)**				<0.001
**Never**	3267 (68.8)	667 (31.1)	2600 (99.9)	
**Former**	221 (4.7)	220 (10.3)	1 (0.04)	
**Current**	1260 (26.5)	1258 (58.6)	2 (0.1)	
**Current drinker, n (%)**	885 (18.6)	867 (40.4)	18 (0.7)	<0.001
**Education, n (%)**				<0.001
**Illiterate**	659 (13.9)	159 (7.4)	500 (19.2)	
**Primary School**	2420 (51.0)	920 (42.9)	1500 (57.6)	
**Junior high**	1517 (32.0)	951 (44.3)	566 (21.7)	
**Senior high**	152 (3.2)	115 (5.4)	37 (1.4)	
**Physical activity, n (%)**				<0.001
**Low**	903 (19.0)	315 (14.7)	588 (22.6)	
**Moderate**	3410 (71.8)	1601 (74.6)	1809 (69.5)	
**High**	435 (9.2)	229 (10.7)	206 (7.9)	
**Hypertension, n (%)**	2295 (48.3)	970 (45.2)	1325 (50.9)	<0.001
**Diabetes, n (%)**	305 (6.4)	107 (5.0)	198 (7.6)	<0.001
**CHD, n (%)**	189 (4.0)	78 (3.6)	111 (4.3)	0.271
**Stroke, n (%)**	113 (2.4)	65 (3.0)	48 (1.8)	0.008
**PAD, n (%)**	212 (4.5)	74 (3.4)	138 (5.3)	0.002
**Use of antihypertensive agents, n (%)**	916 (19.3)	320 (14.9)	596 (22.9)	<0.001
**Use of lipid-lowering agents, n (%)**	86 (1.8)	42 (2.0)	44 (1.7)	0.491
**Family history of CHD, n (%)**	318 (6.7)	124 (5.8)	194 (7.5)	0.022

Data are presented as mean±SD, median (interquartile range) or percent. Chi-square test for categorical variables, the unpaired t test or Mann-Whitney U test for continuous variables.

^a^Data on hsCRP was available for 1887 men and 2286 women.

BMI, body mass index; WHR, waist/hip rate; SBP, systolic blood pressure; DBP, diastolic blood pressure; HDL-C, high density lipoprotein cholesterol; LDL-C, low density lipoprotein cholesterol; hsCRP, high sensitivity C-reactive protein; CHD, coronary heart disease; PAD, peripheral arterial disease.

**Fig 2:**
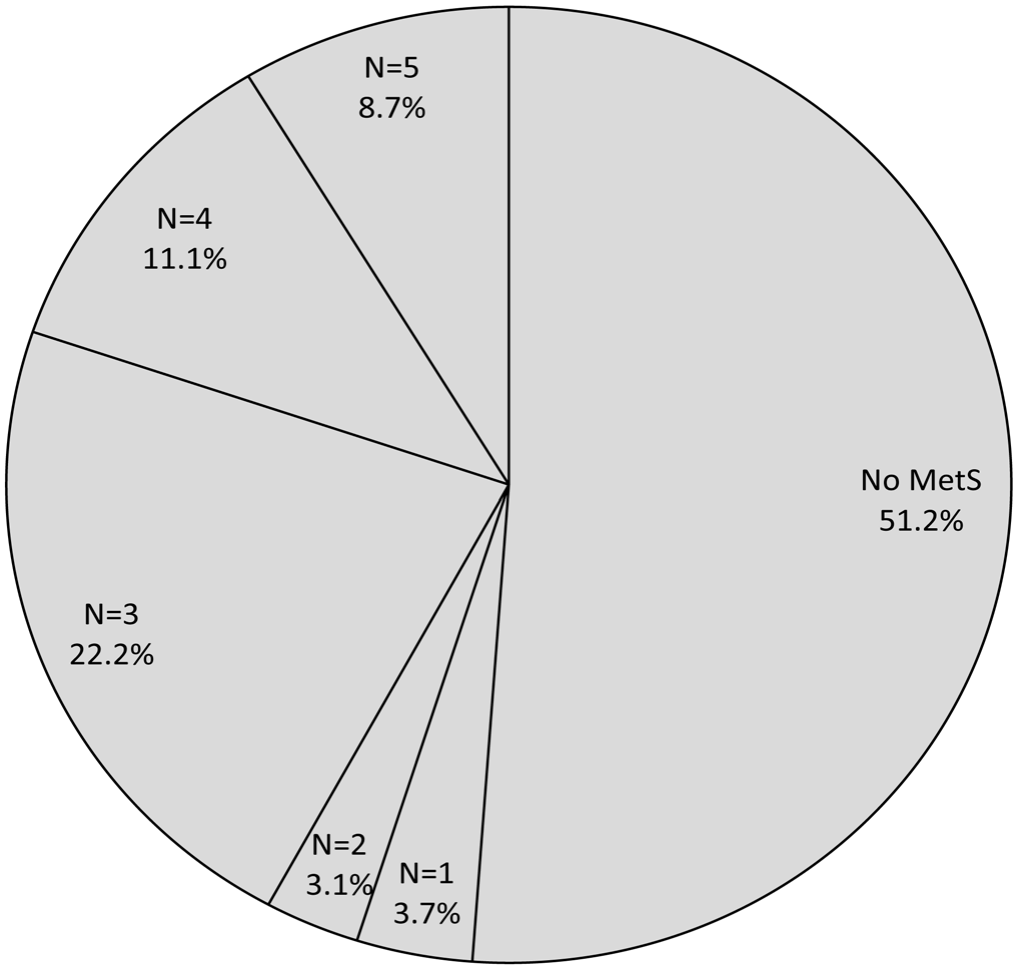
Distribution of participants according to the number of definitions of metabolic syndrome.

Table 3 shows the prevalence of MetS according to different definitions and their individual components in men and women. Women had greater prevalence of individual components of MetS irrespective of the definition used. For men, the MetS definition determined the prevalence. As illustrated in Fig 2, As many as 48.8% of participants met the definition of MetS by any criteria, while only 8.7% of participants had MetS by all five criteria.

**Table 3:** Prevalence (%) of the metabolic syndrome of different definitions of and their individual components in Chinese men and women.

	Total (n = 4748) Prevalence (95% CI)	Men (n = 2145) Prevalence (95% CI)	Women (n = 2603) Prevalence (95% CI)	*P* value
**Full definition of metabolic syndrome**				
**WHO**	13.8 (12.8–14.8)	11.5 (10.2–12.9)	15.7 (14.3–17.1)	<0.001
**CDS**	18.1 (17.0–19.1)	14.8 (13.3–16.3)	20.7 (19.2–22.3)	<0.001
**NCEP-ATPIII updated**	44.3 (42.9–45.8)	32.4 (30.4–34.3)	54.2 (52.3–56.1)	<0.001
**IDF**	40.7 (39.3–42.1)	27.5 (25.6–29.4)	51.5 (49.6–53.4)	<0.001
**JIS**	47.7 (46.2–49.1)	39.7 (37.7–41.8)	54.2 (52.3–56.1)	<0.001
**Elevated blood pressure**				
**CDS, WHO: blood pressure ≥140/90 mmHg or treated**	48.3 (46.9–49.8)	45.2 (43.1–47.3)	50.9 (49.0–52.8)	<0.001
**JIS, NCEP-ATPIII updated, IDF: blood pressure ≥130/85 mmHg or treated**	64.2 (62.9–65.6)	63.2 (61.2–65.3)	65.1 (63.3–66.9)	0.183
**Dyslipidemia**				
**CDS, WHO: TGs ≥1.7 mmol/L and/or HDL-C <0.90 mmol/L in men and <1.03 mmol/L in women**	33.7 (32.4–35.1)	31.9 (30.0–33.9)	35.2 (33.4–37.0)	0.018
**JIS, NCEP-ATPIII updated, IDF: TGs ≥1.7 mmol/L**	29.3 (28.0–30.6)	28.1 (26.2–30.0)	30.4 (28.6–32.1)	0.092
**JIS, NCEP-ATPIII updated, IDF: HDL-C <1.03 mmol/L in men or 1.30 mmol/L in women**	35.8 (34.4–37.1)	18.3 (16.6–19.9)	50.2 (48.3–52.1)	<0.001
**Dysglycemia**				
**CDS, WHO: FPG ≥6.1 mmol/L or known treatment for diabetes**	19.7 (18.6–20.9)	18.4 (16.8–20.1)	20.8 (19.2–22.3)	0.041
**JIS, NCEP-ATPIII updated, IDF: FPG ≥5.6 mmol/L or known treatment for diabetes**	45.2 (43.8–46.7)	45.2 (43.1–47.3)	45.3 (43.4–47.2)	0.935
**Obesity**				
**WHO: WHR >0.90 in men (>0.85 in women) and/or BMI >30 kg/m** ^**2**^	73.1 (71.8–74.3)	68.1 (66.1–70.1)	77.1 (75.5–78.8)	<0.001
**CDS: BMI >25 kg/m** ^**2**^	40.9 (39.5–42.3)	36.3 (34.3–38.4)	44.8 (42.8–46.7)	<0.001
**NCEP-ATPIII updated, IDF: waist circumference ≥90 cm in men or ≥80 cm in women**	60.9 (59.5–62.2)	43.0 (40.9–45.1)	75.5 (73.9–77.2)	<0.001
**JIS: waist circumference ≥85 cm in men or ≥80 cm in women**	70.2 (68.9–71.5)	63.7 (61.7–65.8)	75.5 (73.9–77.2)	<0.001
**WHO: albumin-to-creatinine ratio ≥30 mg/g**	16.3 (15.3–17.4)	14.0 (12.6–15.5)	18.2 (16.7–19.7)	<0.001

WHO, World Health Organization; NCEP-ATPIII, National Cholesterol Education Program Adult Treatment Panel III; IDF, International Diabetes Federation; CDS, Chinese Diabetes Society; JIS, Joint Interim Statement; WHR, waist/hip rate; BMI, body mass index; HDL-C, high density lipoprotein cholesterol; TGs, triglycerides; FPG, fasting plasma glucose.

Tables 4 and 5 present the associations of MetS (as defined by 5 different sources) and their individual components with CHD, stroke, PAD, and CVD in men and women. For men (Table 4), MetS as defined by WHO and NCEP-ATPIII criteria was significantly associated with CHD, stroke, PAD, and CVD. The CDS definition of MetS was significantly associated with stroke, PAD, and CVD but not with CHD. The IDF definition was significantly associated with CHD, PAD, and CVD but not with stroke. The full JIS definition was significantly related to PAD and CVD but this was modestly significant with CHD or stroke. For women (Table 5), all five definitions of MetS were significantly associated with CHD and stroke, but none was associated with PAD. Furthermore, CVD was significantly associated with the WHO, CDS, and IDF definition of MetS but the JIS and NCEP-ATPIII criteria for women was not associated with CVD.

**Table 4:** Associations of the metabolic syndrome of different definitions and their individual components with cardiovascular disease in Chinese men.

	CHD (n = 78) Odds ratios (95% CI)	Stroke (n = 65) Odds ratios (95% CI)	PAD (n = 74) Odds ratios (95% CI)	CHD or Stroke or PAD (n = 201) Odds ratios (95% CI)
**Full definition of metabolic syndrome**				
**WHO**	1.79 (1.02–3.17)	2.18 (1.20–3.97)	2.25 (1.27–3.99)	2.18 (1.50–3.17)
**CDS**	1.25 (0.69–2.26)	2.20 (1.25–3.89)	2.09 (1.21–3.62)	1.82 (1.26–2.62)
**NCEP-ATPIII updated**	1.61 (1.01–2.58)	1.71 (1.02–2.84)	1.92 (1.20–3.09)	1.68 (1.24–2.28)
**IDF**	1.84 (1.14–2.96)	1.30 (0.77–2.23)	1.85 (1.14–3.00)	1.68 (1.23–2.30)
**JIS**	1.53 (0.96–2.43)	1.61 (0.97–2.68)	2.26 (1.40–3.66)	1.73 (1.28–2.34)
**Elevated blood pressure**				
**CDS, WHO: blood pressure** ≥**140/90 mmHg or treated**	1.06 (0.65–1.73)	2.21 (1.24–3.94)	1.15 (0.70–1.90)	1.27 (0.93–1.74)
**JIS, NCEP-ATPIII updated, IDF: blood pressure** ≥**130/85 mmHg or treated**	0.93 (0.55–1.58)	1.95 (0.98–3.87)	1.29 (0.75–2.22)	1.22 (0.86–1.73)
**Dyslipidemia**				
**CDS, WHO: TGs** ≥**1.7 mmol/L and/or HDL-C <0.90 mmol/L in men and <1.03 mmol/L in women**	0.90 (0.53–1.53)	2.04 (1.21–3.43)	1.56 (0.96–2.54)	1.37 (1.00–1.89)
**JIS, NCEP-ATPIII updated, IDF: TGs** ≥**1.7 mmol/L**	0.68 (0.37–1.23)	2.11 (1.24–3.59)	1.75 (1.06–2.87)	1.34 (0.96–1.87)
**JIS, NCEP-ATPIII updated, IDF: HDL-C <1.03 mmol/L in men or 1.30 mmol/L in women**	1.83 (1.08–3.12)	2.26 (1.30–3.96)	1.03 (0.56–1.88)	1.62 (1.13–2.30)
**Dysglycemia**				
**CDS, WHO: FPG** ≥**6.1 mmol/L or known treatment for diabetes**	1.35 (0.80–2.30)	1.79 (1.03–3.10)	1.61 (0.94–2.74)	1.62 (1.15–2.27)
**JIS, NCEP-ATPIII updated, IDF: FPG** ≥**5.6 mmol/L or known treatment for diabetes**	1.07 (0.68–1.70)	1.17 (0.71–1.94)	1.83 (1.13–2.94)	1.30 (0.97–1.75)
**Obesity**				
**WHO: WHR >0.90 in men (>0.85 in women) and/or BMI >30 kg/m** ^**2**^	1.09 (0.65–1.82)	1.10 (0.63–1.92)	2.20 (1.20–4.06)	1.41 (1.00–1.99)
**CDS: BMI >25 kg/m** ^**2**^	1.66 (1.04–2.65)	1.75 (1.05–2.91)	1.74 (1.08–2.79)	1.72 (1.27–2.33)
**NCEP-ATPIII updated, IDF: waist circumference** ≥**90 cm in men or** ≥**80 cm in women**	1.85 (1.15–2.97)	1.24 (0.74–2.06)	1.88 (1.16–3.03)	1.69 (1.25–2.28)
**JIS: waist circumference** ≥**85 cm in men or** ≥**80 cm in women**	1.61 (0.94–2.76)	1.16 (0.67–2.00)	2.07 (1.18–3.65)	1.63 (1.16–2.28)
**WHO: albumin-to-creatinine ratio** ≥**30 mg/g**	1.04 (0.55–1.97)	0.83 (0.40–1.72)	1.49 (0.83–2.68)	1.02 (0.68–1.54)

Adjusted for age, education, physical exercise, smoking, alcohol use, family history of coronary heart disease.

WHO, World Health Organization; NCEP-ATPIII, National Cholesterol Education Program Adult Treatment Panel III; IDF, International Diabetes Federation; CDS, Chinese Diabetes Society; JIS, Joint Interim Statement; WHR, waist/hip rate; BMI, body mass index; HDL-C, high density lipoprotein cholesterol; TGs, triglycerides; FPG, fasting plasma glucose; CHD, coronary heart disease; PAD, peripheral arterial disease.

**Table 5:** Associations of the metabolic syndrome of different definitions and their individual components and cardiovascular disease in Chinese women.

	CHD (n = 111) Odds ratios (95% CI)	Stroke (n = 48) Odds ratios (95% CI)	PAD (n = 138) Odds ratios (95% CI)	CHD or Stroke or PAD (n = 284) Odds ratios (95% CI)
**Full definition of metabolic syndrome**				
**WHO**	1.92 (1.25–2.96)	2.48 (1.35–4.55)	1.19 (0.74–1.91)	1.63 (1.20–2.21)
**CDS**	1.79 (1.18–2.69)	2.66 (1.47–4.81)	0.98 (0.63–1.53)	1.48 (1.11–1.97)
**NCEP-ATPIII updated**	1.69 (1.08–2.64)	2.51 (1.19–5.32)	0.98 (0.68–1.41)	1.28 (0.98–1.68)
**IDF**	1.85 (1.19–2.88)	2.49 (1.21–5.11)	1.02 (0.71–1.47)	1.36 (1.04–1.77)
**JIS**	1.69 (1.08–2.64)	2.51 (1.19–5.32)	0.98 (0.68–1.41)	1.28 (0.98–1.68)
**Elevated blood pressure**				
**CDS, WHO: blood pressure** ≥**140/90 mmHg or treated**	1.23 (0.79–1.90)	3.11 (1.40–6.93)	0.53 (0.37–0.79)	0.89 (0.68–1.17)
**JIS, NCEP-ATPIII updated, IDF: blood pressure** ≥**130/85 mmHg or treated**	1.12 (0.68–1.83)	8.08 (1.91–34.22)	0.59 (0.40–0.86)	0.87 (0.65–1.16)
**Dyslipidemia**				
**CDS, WHO: TGs** ≥**1.7 mmol/L and/or HDL-C <0.90 mmol/L in men and <1.03 mmol/L in women**	1.30 (0.87–1.92)	1.31 (0.73–2.35)	1.02 (0.71–1.46)	1.17 (0.90–1.51)
**JIS, NCEP-ATPIII updated, IDF: TGs** ≥**1.7 mmol/L**	1.42 (0.95–2.11)	1.40 (0.78–2.53)	1.01 (0.69–1.48)	1.21 (0.93–1.58)
**JIS, NCEP-ATPIII updated, IDF: HDL-C <1.03 mmol/L in men or 1.30 mmol/L in women**	1.36 (0.92–2.01)	1.32 (0.74–2.38)	0.98 (0.70–1.39)	1.15 (0.90–1.48)
**Dysglycemia**				
**CDS, WHO: FPG** ≥**6.1 mmol/L or known treatment for diabetes**	1.46 (0.95–2.23)	2.46 (1.36–4.43)	1.33 (0.88–2.00)	1.52 (1.15–2.02)
**JIS, NCEP-ATPIII updated, IDF: FPG** ≥**5.6 mmol/L or known treatment for diabetes**	1.39 (0.93–2.08)	1.53 (0.84–2.80)	1.49 (1.04–2.12)	1.39 (1.08–1.80)
**Obesity**				
**WHO: WHR >0.90 in men (>0.85 in women) and/or BMI >30 kg/m** ^**2**^	1.37 (0.79–2.38)	1.86 (0.73–4.77)	1.38 (0.88–2.17)	1.40 (0.99–1.96)
**CDS: BMI >25 kg/m** ^**2**^	1.38 (0.93–2.04)	1.26 (0.71–2.24)	1.44 (1.02–2.04)	1.35 (1.05–1.73)
**NCEP-ATPIII updated, IDF: waist circumference** ≥**90 cm in men or** ≥**80 cm in women**	1.53 (0.87–2.69)	2.07 (0.81–5.31)	1.26 (0.82–1.94)	1.35 (0.97–1.88)
**JIS: waist circumference** ≥**85 cm in men or** ≥**80 cm in women**	1.53 (0.87–2.69)	2.07 (0.81–5.31)	1.26 (0.82–1.94)	1.35 (0.97–1.88)
**WHO: albumin-to-creatinine ratio** ≥**30 mg/g**	1.44 (0.93–2.25)	0.70 (0.32–1.52)	1.10 (0.71–1.72)	1.16 (0.85–1.58)

Adjusted for age, education, physical exercise, smoking, alcohol use, family history of coronary heart disease.

WHO, World Health Organization; NCEP-ATPIII, National Cholesterol Education Program Adult Treatment Panel III; IDF, International Diabetes Federation; CDS, Chinese Diabetes Society; JIS, Joint Interim Statement; WHR, waist/hip rate; BMI, body mass index; HDL-C, high density lipoprotein cholesterol; TGs, triglycerides; FPG, fasting plasma glucose; CHD, coronary heart disease; PAD, peripheral arterial disease.

In men, low HDL-C, the CDS obesity and IDF/updated NCEP-ATPIII definition of obesity were significantly related to CHD, but no definition of MetS in women was significantly associated with CHD. Elevated blood pressure and CDS/WHO dysglycemia were significantly related to stroke in both men and women. In addition, all dyslipidemic indices were significantly associated with stroke in men, but not in women. All five MetS definitions and most individual components were significantly related to PAD except for elevated blood pressure and ACR in men (Table 4). However, neither a full definition nor its single component was associated with PAD except for dysglycemia (FPG ≥5.6 mmol/L or known treatment for diabetes) and the CDS definition of obesity (BMI >25 kg/m^2^) in women (Table 5).

When diabetic patients were excluded from the analyses, CHD was modestly associated with the WHO, IDF, JIS and updated NCEP-ATPIII criteria and PAD was significantly associated with all five definitions. ORs for stroke ceased to be associated all five MetS definitions for men (S1 Table) but this was not true for women. ORs for CHD were also no longer associated with the five MetS definitions (S2 Table).

## Discussion

In this rural Chinese population, the prevalence of MetS ranged from 13.8% (WHO definition) to 47.7% (JIS definition). As many as 48.8% of participants met the definition of MetS by any criteria, while only 8.7% of participants had MetS by all five criteria. Thus, the identified high-risk population was different across the five MetS definitions. In addition, the new JIS, IDF, and CDS criteria are not more suitable than other definitions for screening high-risk individuals and estimating the risk of CVD from MetS, especially for men. Also, some individual components of MetS are more important determinants of stroke or PAD than the definitions of MetS for women.

The prevalence of MetS according to the different definitions has been reported in Western and Chinese populations [14–16, 18, 19, 24, 25, 27, 32, 33]. Several studies [14–16, 19] in Europe and the US confirmed that the prevalence of MetS was higher using the IDF definition. However, data from China indicated that the prevalence of MetS was higher using the NCEP-ATPIII definition. In a cross-sectional study among a nationally representative sample of 15,838 Chinese adults (35–74 years-of-age) in 2000 to 2001 [27], the overall age-standardized prevalence of MetS according to IDF and the revised-ATP III definitions was 16.5% and 23.3%, respectively. Furthermore, in the China Health and Nutrition Survey [33] conducted in 2009, which included 7,488 Chinese adults aged ≥18 years, the overall age-standardized prevalence of MetS was 21.3%, 18.2%, and 10.5% based on the definitions of revised NCEP-ATPIII, IDF and CDS criteria, respectively.

In our study, we noted a prevalence of MetS that was higher than those of previous studies and which varied significantly according to how it was defined. The prevalence of MetS was higher according to the JIS and updated NCEP-ATPIII definitions, and the number of individuals identified by the JIS or updated NCEP-ATPIII definition as having MetS exceeded those identified by WHO or CDS definitions by three times. These results suggest that the prevalence of MetS varies according to how it is defined and the population composition (age, gender, race and ethnicity) [34]. Also, the prevalence of abdominal obesity, which was higher in Western populations, and the requirement of insulin resistance or abdominal obesity according to WHO or IDF definitions offers conservative estimates for the prevalence of MetS in Chinese populations.

In our study, the prevalence of MetS according to all five definitions was higher in women than in men, and these data are similar to those form other Chinese populations [24, 25, 27, 33]. Women had a significantly higher prevalence of central obesity and reduced HDL-cholesterol compared with men [25].

To date, several prospective studies conducted in Western populations have compared the associations of different MetS definitions and cardiovascular events, but these results were different than ours. In a British women’s heart and health study [14], which involved a random sample of 3,589 British women aged 60 to 79 years, the prevalence of MetS was significantly higher using the IDF definition, but there was no suggestion that this definition predicted CHD risk better than the WHO or NCEP definitions. Furthermore, a population-based cohort study of non-diabetic Swedish adults, 46–68 years-of-age [16] revealed that the prevalence of MetS according to the IDF definition was higher than that as defined by the NCEP-ATPIII definition, but the IDF definition was not superior to the NCEP-ATPIII definition for predicting cardiac events and stroke. Also, the NCEP, but not the IDF definition, was associated with increased risk of stroke after adjusting for age, sex and other risk factors. However, A collaborative data analysis [18] based on 6 Finnish and Swedish cohorts, 25–74 years-of-age indicated that the WHO, NCEP, revised NCEP, and IDF definitions of MetS predicted the incidence of CHD equally well in men, but the IDF definition performed better in women. In a non-diabetic elderly US population [19], the modified WHO, IDF and NCEP/AHA definitions of MetS were significantly associated with coronary events and total cardiovascular events, but only the modified WHO definition was associated with risk of cerebrovascular events.

In China, He’s group [25] reported that the prevalence of MetS was higher according to IDF than the original NCEP definition and that the IDF criteria appeared to be better for screening and estimating risk of MetS in elderly Chinese people. However, we found that all five definitions of MetS were associated with CHD and stroke equally well in women, but the WHO and updated NCEP-ATPIII definitions performed better in men. Interestingly, our study data agreed with those from a cohort study from Thailand [17] in which the revised NCEP-ATPIII definition, using the Asian cutoff for WC, produced a higher prevalence of MetS and a stronger association with both CVD and all-cause mortality compared with the IDF definition. The discrepancy between our study and other Chinese and Western population studies may be due to the differences in the demographics of the population studied and the ethnic-specific cutoff for WC used in the MetS criteria.

To date, several studies in Western populations showed that MetS was associated with PAD [11–13]. Furthermore, Conen’s group [12] reported that MetS is associated with an increased risk of PAD and this risk was completely attenuated by CRP and soluble intercellular adhesion molecule-1. In China, He and coworkers [25] reported that MetS was significantly associated with PAD after adjustment for potential confounders, but gender difference and whether the association was related to CRP were not explored in that study. In our study, we found that all five definitions of MetS were significantly related to PAD in men, but neither a full definition nor an individual component except for dysglycemia (FPG ≥5.6 mmol/L or known treatment for diabetes) and CDS obesity (BMI >25 kg/m^2^) was associated with PAD in women after adjustment for potential confounders. Further adjustment for CRP, the association of MetS and PAD was not substantially decreased in men (S3 Table), although our previous studies proved that CRP was associated with MetS and type 2 diabetes in this rural Chinese population [35, 36]. The significant gender difference in the association of MetS and PAD may be due to dysglycemia, obesity, and lifestyle; we observed that women had a significantly greater prevalence of diabetes (7.6% versus 5.0%) and obesity (44.8% versus 36.3%) than men, and that men had a significantly higher rate of current smoking (58.6% versus 0.1%) and alcohol consumption (40.4% versus 0.7%) than women.

In our study, most of the individual components of MetS are associated with CHD, stroke, and PAD as equally well as its full definition in men. However, high blood pressure or high FPG are more important determinants of stroke or PAD than any definition of MetS in women. Possibly, MetS inevitably represents a heterogeneous group with the overall effect estimate being a weighted average of some very high-risk individuals and some who are lower risk [14].

To our knowledge, this study is the first to investigate the relationship of CVD and all five MetS definitions, which have been proposed for screening high-risk individuals in Chinese population. In addition, a strict quality control program was implemented in the study to ensure data reliability and validity. Most importantly, the present study evaluated a rural Chinese population from China, where about half of Chinese people live in rural districts and have different lifestyles, diets, and socioeconomic position, compared to people living in urban areas. Our data suggest that in rural China, the prevalence of MetS and its components are higher in women than in men, and the risks for CHD, stroke, and PAD are higher in participants with MetS than in those without MetS. Therefore, it is necessary to develop effective strategies to prevent and treat both MetS and its individual components in rural China, especially in women.

Limitations of this study must be noted. First, it is possible that exclusions may lead to selection bias since 2,082 persons were excluded from a total of 6,830 participants who took part in the HES. Second, our findings must be interpreted with caution because this cross-sectional analysis data could not be used to investigate the association between MetS and new CHD, stroke or PAD cases. Furthermore, with a cross-sectional study we obtain all case at a point in time, these include older cases having survived to this point in time plus more recent new cases. If MetS is strongly associated with fatal CHD, stroke or PAD, then these cases are absent at this point in time and an underestimate of association is possible. Lastly, the diagnosis of CHD and stroke in the present analysis are based on interview questions and are therefore subject to potential errors and biases related to recall.

In conclusion, high-risk population are identified uniquely according to different MetS definitions and the new JIS, IDF, and CDS criteria may not be more suitable than WHO and updated NCEP-ATPIII definitions for screening high-risk individuals and estimating the risk of CVD from MetS, especially in rural Chinese men. Also, some individual components of MetS are more important determinants of stroke or PAD than any definition in rural Chinese women. Further prospective studies should determine the associations of different MetS definitions and new CVD events and establish WC cutoff points for CVD risk estimation. These studies should also explore whether individual components of MetS are more important determinants of CVD than any full definitions of MetS in a Chinese population. In addition, our data indicate that MetS and its components, such as hypertension, high FPG, and central obesity, are serious public health problems, and effective public health strategies for preventing and treating MetS and its components should be developed to reduce social and medical burden in rural China.

## Supporting Information

S1 Table Associations of the metabolic syndrome of different definitions with CHD, stroke, and PAD in Chinese non-diabetic men. (XLSX)

S2 Table Associations of the metabolic syndrome of different definitions with CHD, stroke, and PAD in Chinese non-diabetic women. (XLSX)

S3 Table Associations of the metabolic syndrome of different definitions with peripheral arterial disease (PAD) in Chinese men and women. (XLSX)
